# Eduardo Slatopolsky: A Pioneer in the Field of Mineral and Bone Disorders in Kidney Disease

**DOI:** 10.7759/cureus.68543

**Published:** 2024-09-03

**Authors:** Mohammad Tinawi

**Affiliations:** 1 Nephrology, Nephrology Specialists, Munster, USA

**Keywords:** clinical nephrology, internal medicine, metabolic bone diseases, legacy, lives and achievements of medical pioneers, historical vignette

## Abstract

Eduardo Alberto Slatopolsky (1934-2024), Washington University Joseph Friedman Professor Emeritus of Medicine, a prominent physician-scientist, was born in Buenos Aires, Argentina. The impact of his research in the area of mineral and bone disorders in kidney disease has been profound for over 50 years starting in the 1960s. He was a global authority on secondary hyperparathyroidism. He was instrumental in illustrating the role of hyperphosphatemia in chronic kidney disease (CKD) and in developing the first reliable parathyroid hormone (PTH) assay. His research led to the utilization of calcium salts as phosphate binders replacing the toxic aluminum salts. Moreover, he illustrated the role of vascular calcifications in CKD patients, paving the way for non-calcium phosphate binders. He proposed the use of calcitriol and later vitamin D analogs in the management of secondary hyperparathyroidism, which is the current standard of care. He demonstrated the decreased expression of the calcium-sensing receptor in parathyroid tissue in CKD patients. Dr. Slatopolsky's global legacy will last for generations.

## Introduction and background

Eduardo Alberto Slatopolsky (Eduardo or Ed to his friends, colleagues, and coworkers), Washington University Joseph Friedman Professor Emeritus of Medicine, was born in Buenos Aires, Argentina, on December 12, 1934. He earned a Bachelor of Science degree from Nicolás Avellaneda National College in 1952. He graduated with a medical degree from the University of Buenos Aires in 1959. He became fascinated with renal physiology and decided to pursue a career in nephrology in his second year of medical school [1]. He started his residency in Cleveland, Ohio, after he moved to the United States in 1960. He had the opportunity to work with the inventor and father of hemodialysis, Dr. Willem Kolff, while doing a rotation at the Cleveland Clinic. In 1963, he started his nephrology fellowship under the direction of Dr. Neil Bricker, the first Chief of the Nephrology Division at Washington University School of Medicine in Saint Louis, Missouri. While at Washington University, he worked with Dr. Saulo Klahr, another giant in the field of nephrology [2].

**Figure 1:**
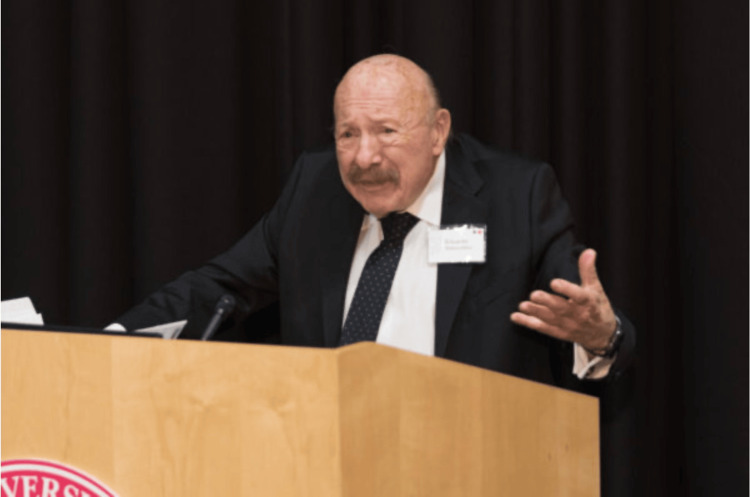
Eduardo Slatopolsky, MD Image Credit: Cynthia Ritter, Medical Writer/Internet Content Editor, Division of Nephrology, Washington University School of Medicine

**Figure 2:**
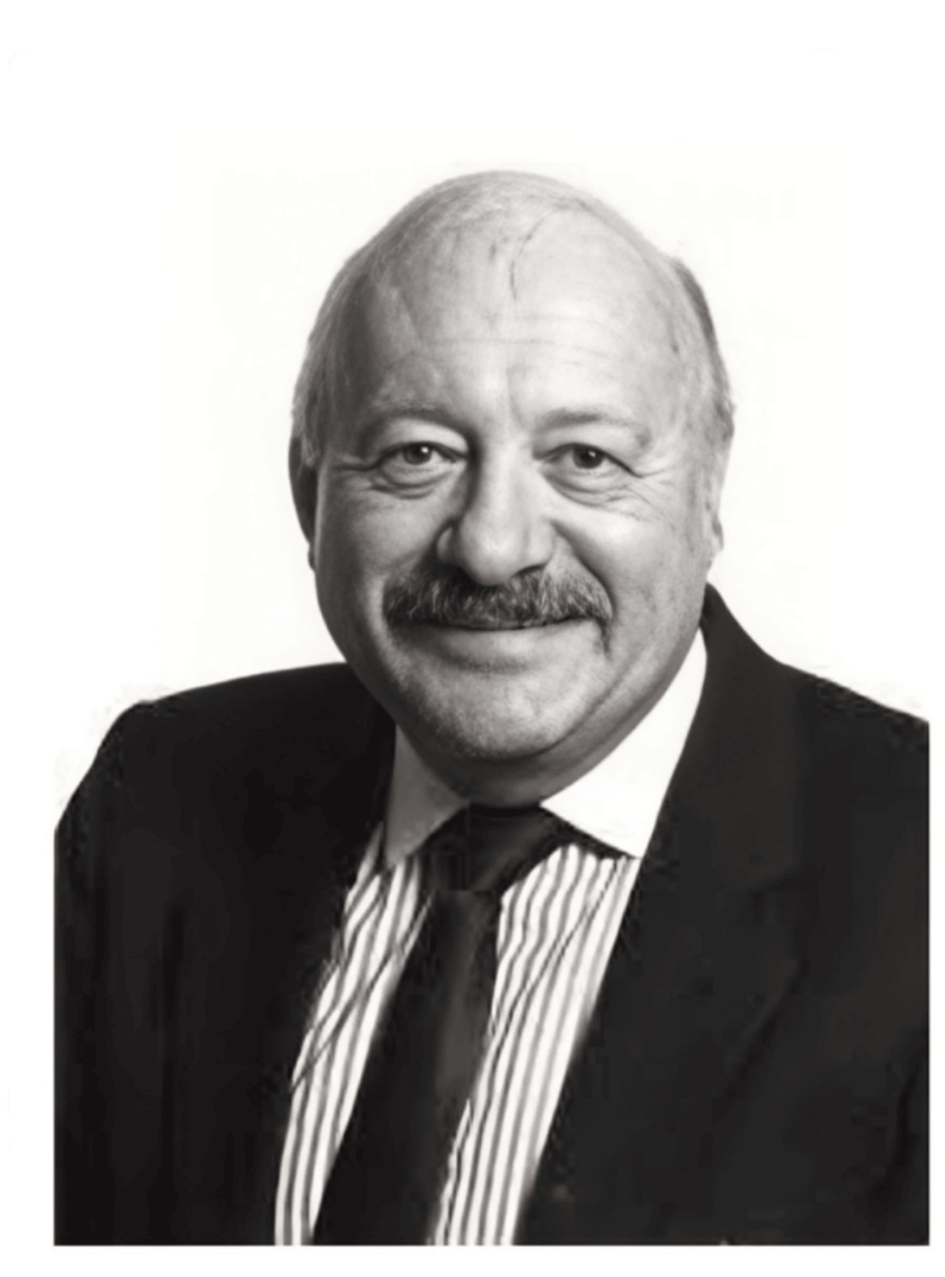
Eduardo Slatopolsky, MD Image Credit: Victoria Fraser, MD, Washington University School of Medicine

Dr. Slatopolsky spent his fruitful career as a physician-scientist at Washington University School of Medicine, focusing on the interaction of mineral metabolism and kidney disease (Figure 1 and Figure 2). He became a Professor of Medicine in 1975, and his distinguished career spanned over 50 years. He mentored generations of physicians and scientists. He founded the Chromalloy Dialysis Center at Washington University and was the director of the Parathyroid Hormone-Vitamin D Laboratory and the director of the Chromalloy American Kidney Center until his retirement in 2016. Eduardo was known for his great personality, sense of humor, and great dance moves [1]. According to ResearchGate.com, he had 429 research items including numerous original papers and book chapters, amassing over 29,500 citations. He gave numerous lectures on national and international levels. He earned numerous accolades from scientific societies, including the prestigious Belding H. Scribner Award of the American Society of Nephrology, the Lifetime Achievement "Master Physician" Award bestowed by Washington University School of Medicine/Barnes-Jewish Hospital, and the Amgen International Award of the International Society of Nephrology.

Dr. Slatopolsky died on April 24, 2024, in Saint Louis, Missouri, at the age of 89. He was the devoted husband of the late Judith Miriam Hirshfeld for 52 years. He is survived by three children, six grandchildren, and one great-granddaughter.

When I was a renal fellow at Saint Louis University Hospital in the mid-1990s, I had the good fortune of meeting Dr. Slatopolsky when he was invited by our renal division chief Dr. Kevin Martin to give us a lecture. Dr. Martin himself collaborated with Dr. Slatopolsky on numerous research projects in the area of bone and mineral disorders over decades. I found Dr. Slatopolsky witty, funny, and extremely knowledgeable. Over the years, I attended several lectures given by Dr. Slatopolsky at premier renal meetings. He always showed complicated basic science slides, followed by his reassurance to the audience: "Don't worry, I will be easy on you!," and then he proceeded to explain and simplify. The last time I met Dr. Slatopolsky was over a decade ago, I approached him and congratulated him on a great lecture, and he gave me a big smile and said: "you are too kind."

## Review

The focus of this review is on the distinguished scientific career of Dr. Slatopolsky. This is not a review of current literature on mineral and bone disorders in CKD; rather, it is a scientific testimony to the achievements of a notable individual in renal medicine. It is imperative to emphasize that medical research is a teamwork and not a one-man show. Therefore, a tremendous and a well-deserved credit goes to everyone who worked at the Parathyroid Hormone-Vitamin D Laboratory at Washington University and to all the collaborators from other centers around the world.

The syndrome of chronic kidney disease-mineral and bone disorder (CKD-MBD) is a systemic disorder characterized by one or more of the following abnormalities: abnormalities in bone turnover, mineralization, volume, or strength; vascular or soft tissue calcifications; or abnormal calcium, phosphorus, PTH (an 84-amino acid single-chain polypeptide), or vitamin D metabolism [3]. Elevated PTH in patients with CKD-MBD defines secondary hyperparathyroidism [4]. Dr. Slatopolsky studied phosphate excretion in uremia and became the world-leading authority on secondary hyperparathyroidism in CKD patients [2]. He excelled in basic research, including molecular biology and clinical science. He created physiological models searching for answers from experimental animals to the bedside. His major contributions to science can be divided into the following categories.

The "trade-off" hypothesis

Initially, Dr. Bricker proposed that with loss of renal function (loss of nephrons), the remaining nephrons compensate by increasing function to maintain homeostasis. In the case of phosphorus, the remaining nephrons increase phosphorus excretion. This leads to abnormalities of the uremic state such as secondary hyperparathyroidism [5]. Dr. Slatopolsky's laboratory demonstrated in 1968 that the decrease in phosphate reabsorption is enhanced by the severity of CKD [6].

The "trade-off" hypothesis was proposed by Dr. Bricker and Dr. Slatopolsky. It states that the decline in kidney function in CKD leads to hyperphosphatemia, which in turn stimulates the release of PTH to enhance phosphate excretion [7]. This leads to secondary hyperparathyroidism [8,9]. Drs. Bricker and Slatopolsky showed that with gradual loss of renal function, hyperphosphatemia and hypocalcemia transiently ensue; this leads to a stepwise increase in PTH. This stimulus to PTH release is increased in CKD stages 4 and 5 (GFR <30 ml/min) because hypocalcemia and hyperphosphatemia become persistent in those stages. Moreover, vitamin D resistance leads to hypocalcemia, osteomalacia, and increased PTH release [8]. In dialysis patients, persistent hypocalcemia and hyperphosphatemia lead to osteitis fibrosa which is characterized by elevated PTH level. Dr. Slatopolsky was an authority on the management and risk of hyperphosphatemia in CKD. He emphasized the role of dietary phosphate control and phosphate binders [10-12].

Developing a PTH radioimmunoassay

Accurate diagnosis and treatment monitoring of secondary hyperparathyroidism requires measurement of PTH. This measurement is essential to prevent PTH oversuppression which may lead to increased fracture risk due to adynamic bone disease [13]. Dr. Slatopolsky developed a very sensitive PTH radioimmunoassay to elucidate the pathophysiology of secondary hyperparathyroidism [1,13,14]. This was in the 1970s when PTH measurement was exceedingly challenging. The assay was obtained from a rooster named "Macho" [1,9]. PTH radioimmunoassay results differ depending on the part of the PTH polypeptide targeted by the specific antibody [15]. Currently, second-generation assays are usually used (intact PTH assays). They measure the entire PTH polypeptide chain (amino acids 1-84) without the N-terminal fragments [13].

The role of phosphorus in secondary hyperparathyroidism

Dr. Slatopolsky observed the role of phosphorus retention in triggering secondary hyperparathyroidism in CKD and called for long-term prospective studies on phosphate restriction in CKD patients [7]. This led to the current practice of phosphate restriction in advanced CKD patients [9,16,17]. In 1971, Dr. Slatopolsky studied phosphate clearance in two groups of dogs with declining renal function [9]. In the first group, phosphate intake was 1200 mg/day, and in the second group, it was reduced to less than 100 mg/day. In the first group, phosphate elimination increased, with a 20-fold increase in PTH level. In the second group with phosphate restriction, PTH level and phosphate elimination did not increase. This study showed the effect of phosphate restriction on secondary hyperparathyroidism in advanced kidney disease. Dr. Slatopolsky and collaborators demonstrated the direct action of phosphate on the function of the parathyroid glands in vivo and in vitro. This action was independent of changes in serum-ionized calcium and calcitriol [18]. In 1972, Dr. Slatopolsky and collaborators described tertiary hyperparathyroidism as an autonomous PTH secretion in a minority of CKD patients that is not suppressed by phosphate reduction or calcium elevation [19].

Phosphate binders

Elucidating the role of phosphate restriction in the pathogenesis of secondary hyperparathyroidism led to the utilization of aluminum salts as phosphate binders. Aluminum salts are associated with significant chronic toxicity including osteomalacia, muscle pain, neurotoxicity including dementia and myoclonic jerks, and microcytic anemia. Dr. Slatopolsky recognized the substantial toxicity of aluminum salts [20]. He published the initial study that utilized calcium carbonate as a phosphate binder in dialysis patients in 1986 [21]. The study was done in 20 patients, and calcium carbonate was an effective binder; however, it led to hypercalcemia in most patients. Due to hypercalcemia, calcium in the dialysate was reduced from 3.5 mEq/L (which was the standard at the time) to 2.5 mEq/L. The mean dose of calcium carbonate utilized was high by today's standard (mean dose 8.5 g daily, with a range of 2.5-17 g). In 1992, Dr. Slatopolsky published a study regarding the use of calcium acetate as a phosphate binder in dialysis patients [22]. This study also included 20 patients, and it showed that calcium acetate controlled phosphorus, calcium, and PTH equally well as calcium carbonate but with one-half the dose of elemental calcium. Later, he was instrumental in defining the role of calcium binders in vascular calcification, leading to the utilization of sevelamer, a non-calcium phosphate binder, years later. The RenaGel Study was published in 1999 and included 172 hemodialysis patients. Sevelamer (a nonabsorbed phosphate binder that is calcium-free and aluminum-free) was given for eight weeks. Sevelamer controlled serum phosphorus and lowered PTH and total cholesterol levels without inducing hypercalcemia [23]. 

The role of calcitriol in secondary hyperparathyroidism

In a landmark paper in 1984, Dr. Slatopolsky demonstrated marked PTH suppression by intravenous calcitriol in uremic patients [24]. He illustrated the role of intravenous calcitriol as it allows greater delivery to target tissues compared with oral calcitriol. Later, he studied vitamin D analogs such as paricalcitol and their role in the management of secondary hyperparathyroidism [25,26]. Paricalcitol was about 10 times less likely to release skeletal calcium and phosphorus compared to calcitriol. The initial study was done in parahyroidectomized rats [25]. The mechanism of the lower calcemic effect of paricalcitol is an acquired post-receptor resistance at the bone and intestinal levels [26]. The use of oral and intravenous calcitriol and vitamin D analogs became instrumental in the management of secondary hyperparathyroidism. 

The role of calcium-sensing and vitamin D receptors in secondary hyperparathyroidism

Dr. Slatopolsky and collaborators demonstrated the decreased expression of the calcium-sensing receptor in parathyroid tissue and that dietary phosphate restriction prevents both decreased calcium-sensing receptor expression and parathyroid hyperplasia in kidney failure [27]. Dr. Slatopolsky illustrated the role of vitamin D in calcium and bone metabolism and the malfunction of the vitamin D receptor in secondary hyperparathyroidism. He emphasized the hormonal nature of vitamin D and its active metabolites [28]. Calcium-sensing receptor expression in parathyroid tissue is restored upon reversal of secondary hyperparathyroidism due to phosphorus restriction [12].

## Conclusions

Dr. Slatopolsky left an indelible mark and a lasting legacy in the field of nephrology. He will be missed by legions of nephrologists and scientists worldwide for years to come. His contributions to the field of nephrology and in particular metabolic bone disease continue to have a profound impact on the lives of countless renal patients.
